# Overexpression of LINC00607 inhibits cell growth and aggressiveness by regulating the miR-1289/EFNA5 axis in non-small-cell lung cancer

**DOI:** 10.1515/med-2023-0649

**Published:** 2023-06-12

**Authors:** Li Zhang, Huimin Liu, Yan Long, Yuan Zhang

**Affiliations:** Department of Clinical Laboratory Center, The First Affiliated Hospital of Xinjiang Medical University, Urumqi 830011, Xinjiang, China; Department of Clinical Laboratory Center, Hospital of Xinjiang Production and Construction Corps, The Second Affiliated Hospital of Medical College of Shihezi University, Urumqi 830092, Xinjiang, China; Department of First Aid Center, Hospital of Xinjiang Production and Construction Corps, The Second Affiliated Hospital of Medical College of Shihezi University, Urumqi 830092, Xinjiang, China; Department of Clinical Laboratory Center, Cancer Hospital of Xinjiang Medical University, No. 789, Suzhou East Street, Xinshi District, Urumqi 830000, Xinjiang, China

**Keywords:** non-small-cell lung cancer, LINC00607, miR-1289, EFNA5

## Abstract

Long non-coding RNAs (lncRNAs) play a key role in cancer progression, including non-small-cell lung cancer (NSCLC). LncRNA long intergenic non-protein-coding RNA 00607 (LINC00607) was previously discovered to be downregulated in lung adenocarcinoma tissues. Nevertheless, the potential role of LINC00607 in NSCLC is still unclear. The expression of LINC00607, miR-1289, and ephrin A5 (EFNA5) in NSCLC tissues and cells was tested by reverse transcription quantitative polymerase chain reaction. Cell viability, proliferation, migration, and invasion were measured by 3-(4,5-dimethylthiazole-2-y1)-2,5-diphenyl tetrazolium bromide, colony formation, wound healing, and Transwell assays. The relationship among LINC00607, miR-1289, and EFNA5 in NSCLC cells was verified by the luciferase reporter assay, RNA pull-down assay, and RNA immunoprecipitation assay. In this study, LINC00607 was downregulated in NSCLC, and its low level is associated with poor prognosis of NSCLC patients. Furthermore, LINC00607 overexpression repressed NSCLC cell viability, proliferation, migration, and invasion. LINC00607 bound with miR-1289 in NSCLC. EFNA5 was a downstream target of miR-1289. EFNA5 overexpression also inhibited NSCLC cell viability, proliferation, migration, and invasion. EFNA5 knockdown antagonized the influence of LINC00607 overexpression on NSCLC cell phenotypes. Overall, LINC00607 serves as a tumor suppressor gene in NSCLC through binding with miR-1289 and modulating the level of EFNA5.

## 1 Introduction

Lung cancer (LC) is a common cancer worldwide, with approximately 1.6 million newly diagnosed cases each year [1]. Non-small-cell lung cancer (NSCLC) accounts for 75–80% of all LCs [2]. Despite our increasing understanding of pathogenesis, immunologic control, and treatment options for NSCLC, NSCLC is still recognized as the main reason of cancer-related mortality [3]. Faced with the discouraging situation, we are aware of the urgency to identify potentially effective biomarkers and understand the underlying molecular mechanism, thus contributing to the improvement of NSCLC therapy.

Long non-coding RNAs (lncRNAs), with a length of over 200 nucleic acids, belong to non-coding RNAs [4]. LncRNAs are implicated in transcriptional, post-transcriptional, and translational regulation and play crucial roles in different biological processes, involving cell growth, cell metabolism, and cell differentiation [5]. Furthermore, lncRNAs usually function as competing endogenous RNAs, binding competitively to microRNAs (miRNAs) to affect the regulation of miRNAs on the downward target expression [6]. Increasing studies have shown that lncRNAs act as effective regulators in cancer development and metastasis [7]. The significant role of lncRNAs in NSCLC has been discussed in many studies. For example, lncRNA small nucleolar RNA host gene 1 contributes to enhanced NSCLC cell growth, migration, and invasion via upregulating metadherin by sponging miR-145–5p [8]. LncRNA fetal-lethal non-coding developmental regulatory RNA promotes cell proliferation and aggressiveness in NSCLC by negatively interacting with miR-761 and regulating tissue inhibitor of metalloproteinase-2 expression [9]. In addition, upregulated lncRNA musculoaponeurotic fibrosarcoma oncogene family, protein G antisense 1, promotes cell growth and motion by modulating the miR-339-5p/matrix metalloproteinase 15 axis in NSCLC [10]. In this case, it is important for us to find potentially effective biomarkers for NSCLC.

Here, we introduced a relatively native lncRNA long intergenic non-protein-coding RNA 00607 (LINC00607), which was found to be downregulated in lung adenocarcinoma (LUAD) tissues [11]. As a species-specific lncRNA, LINC00607 is only found in humans, resulting in little research on its functional mechanism. For example, LINC00607 is upregulated in osteosarcoma and its knockdown suppresses osteosarcoma cell growth, invasion, and migration via completely binding to miR-607 and downregulating E2F transcription factor 6 expression [12]. LINC00607 is upregulated in thyroid cancer, which promotes doxorubicin resistance, growth, and colony formation, and represses cell apoptosis of thyroid cancer cells by targeting caspase 9 [13]. The role of LINC00607 in NSCLC was not investigated in previous studies.

We aimed to explore the potential biological function of LINC00607 and attempted to analyze LINC00607-invovled regulatory mechanism in NSCLC, thus providing a potential novel insight into the therapy of NSCLC.

## 2 Materials and methods

### 2.1 Tissue samples and cell lines

All NSCLC tissues (*n* = 35) and non-tumor tissues (*n* = 35) were obtained at Cancer Hospital of Xinjiang Medical University (Xinjiang, China). All the samples were stored at −80℃ once collected from patients, who have not received anticancer therapy before surgery. This study was conducted after obtaining informed consent from the patients and approval from the Ethics Committee of Cancer Hospital of Xinjiang Medical University (Xinjiang, China).

NSCLC cell lines A549, H460, and H1299 and human normal Beas-2B cell line were all purchased from State Key Laboratory of Oncology in South China and cultured in Roswell Park Memorial Institute-1640 (RPMI-1640; GIBCO, Grand Island, NY, USA) medium containing 10% fetal bovine serum (FBS; GIBCO). The cell lines were incubated in a humidified atmosphere with 5% CO_2_ at 37℃.

### 2.2 Cell transfection

The full length of LINC00607 or ephrin A5 (EFNA5) was inserted into pcDNA3.1 vector to overexpress LINC00607 or EFNA5, with empty pcDNA3.1 vector as the negative control. Short hairpin RNA targeting LINC00607 or EFNA5 (sh-EFNA5) was used to knock down LINC00607 or EFNA5 expression, with sh-NC as the negative control. miR-1289 inhibitor was used to downregulate miR-1289 expression, and NC inhibitor was used as the negative control. NSCLC cells were incubated in 24-well plates until reaching 70–90% monolayer. The aforementioned plasmids were transfected into NSCLC cells using Lipofectamine 3000 reagent (Invitrogen). All the vectors were purchased from GenePharma (Suzhou, China). The transfected cells were harvested after 48 h for the following experiments.

### 2.3 Reverse transcription quantitative polymerase chain reaction (RT-qPCR)

Total RNA was extracted from tissues and cells using TRIzol reagent (Invitrogen). A cDNA Reverse Transcription Kit (Promega, Madison, WT, USA) was used to reversetranscribe 1 μg of total RNA into complementary DNA. Quantitative PCR analysis was conducted using Power SYBR Green PCR Master Mix (Promega). Glyceraldehyde-3-phosphate dehydrogenase (GAPDH) or small nuclear RNA U6 was used, respectively, as the negative controls for lncRNA (and mRNA) or microRNA (miRNA) expression level. The relative expression was calculated using the 2^−ΔΔCt^ method.

### 2.4 3-(4,5-Dimethylthiazole-2-y1)-2,5-diphenyl tetrazolium bromide (MTT) assay

Transfected NSCLC cells were seeded in 96‐well plates (6,000 cells/well), followed by incubation for 0, 24, 48, and 96 h. Next, 10 μL of MTT (Beyotime) was added to each well for 4 h to react with the cells. Subsequently, the supernatant was deserted, and the crystal was dissolved using 150 μL of dimethylsulfoxide (Thermo Fisher Scientific). Finally, the optical density at 490 nm was measured by an EnSpire Multimode Plate Reader (PerkinElmer).

### 2.5 Colony formation assay

The transfected NSCLC cells (1 × 10^4^) were cultured in 6-well plates. Two weeks later, the cells were fixed and dyed in methanol (Sigma-Aldrich) and 0.1% crystal violet (Sigma-Aldrich). Finally, colonies were counted and imaged by a gel documentation system (Bio-Rad, Shanghai, China). Colonies with at least 50 cells were considered significant.

### 2.6 Wound healing assay

Transfected cells (1 × 10^6^) were seeded in 6-well plates and grown until 90% confluence. Then, a 200 μL sterile pipette tip was used to scrape the confluent cell monolayer to create a linear wound. The suspension cells were incubated in Dulbecco’s modified Eagle medium (GIBCO) with free FBS after being washed twice with phosphate-buffered saline (Invitrogen). The wound was observed regularly and imaged after 24 h.

### 2.7 Transwell assay

The upper side of 24-well Matrigel-precoated transwell chambers (Corning) was added with free-serum medium containing 5 × 10^4^ NSCLC cells. The lower chamber was added with medium with 10% FBS. After 24 h, the invaded cells were fixed by 4% paraformaldehyde and stained with 1% crystal violet. Three random fields were counted in each chamber using an inverted microscope (Olympus).

### 2.8 RNA pull-down assay

LINC00607 and NC were biotin-labeled into Bio-LINC00607 and Bio-NC. Cell lysates and biotinylated RNAs were incubated with streptavidin-coated magnetic beads (Life Technologies) at 4°C for 3 h. Finally, RNA–protein binding mixture was purified and then detected by RT-qPCR.

### 2.9 Luciferase reporter assay

The wild-type (WT) LINC00607 and 3′-untranslated regions of WT EFNA5 were subcloned into pGL3 luciferase vectors (Promega) and named as LINC00607-WT and EFNA5-WT. Their mutant-type (MUT) controls were also constructed by using a Phusion Site-Directed Mutagenesis Kit (Thermo Fisher Scientific) and named as LINC00607-MUT and EFNA5-MUT. LINC00607-WT/MUT or EFNA5-WT/MUT was cotransfected with miR-1289 inhibitor/NC inhibitor into NSCLC cells using Lipofectamine 3000. Luciferase activities were detected after 48 h by a Dual‐Luciferase Reported Assay System (Promega).

### 2.10 RNA immunoprecipitation (RIP) assay

RIP assay was performed using Magna RNA-binding protein immunoprecipitation kit (Millipore). NSCLC cells were lysed in lysis buffer containing protease inhibitor cocktail and RNase inhibitor. Cell lysates were cultured in RIP buffer containing magnetic beads coated with Ago2 antibody, with normal IgG regarded as the control. After 2 h of incubation at 4°C, the coprecipitated RNA was eluted from beads and subjected to RT-qPCR analysis.

### 2.11 Western blot

NSCLC cells were lysed with RIP assay lysis buffer (Sigma-Aldrich) supplemented with protease inhibitor cocktail. A bicinchoninic acid assay kit (Sigma-Aldrich) was used to determine the protein concentration. The proteins were separated by 10% sodium dodecyl sulfate–polyacrylamide gel electrophoresis and transferred to a polyvinylidene fluoride (PVDF) membrane. The primary antibodies against EFNA5 (sc-81945; 1:1,000; Santa Cruz Biotechnology) and GAPDH (ab8245; 1:2,000; Abcam) were cultured with non-fat milk-blocked PVDF membrane overnight at 4℃. The secondary antibody was added for co-culture at indoor temperature for 1 h. Protein bands were analyzed by densitometric analysis.

### 2.12 Statistical analysis

Data from three dependent experiments are shown as mean ± standard deviation and were analyzed using SPSS 21.0 software (IBM Corp., Armonk, NY, USA). Student’s *t*-test or one-way analysis of variance followed by Turkey’s *post hoc* test was used for analyzing the differences between two groups or among multiple groups. *P*-value <0.05 indicates systematic significance.

## 3 Results

### 3.1 LINC00607 is downregulated in NSCLC tissues and cells and associated with a poor prognosis

**Figure 1:**
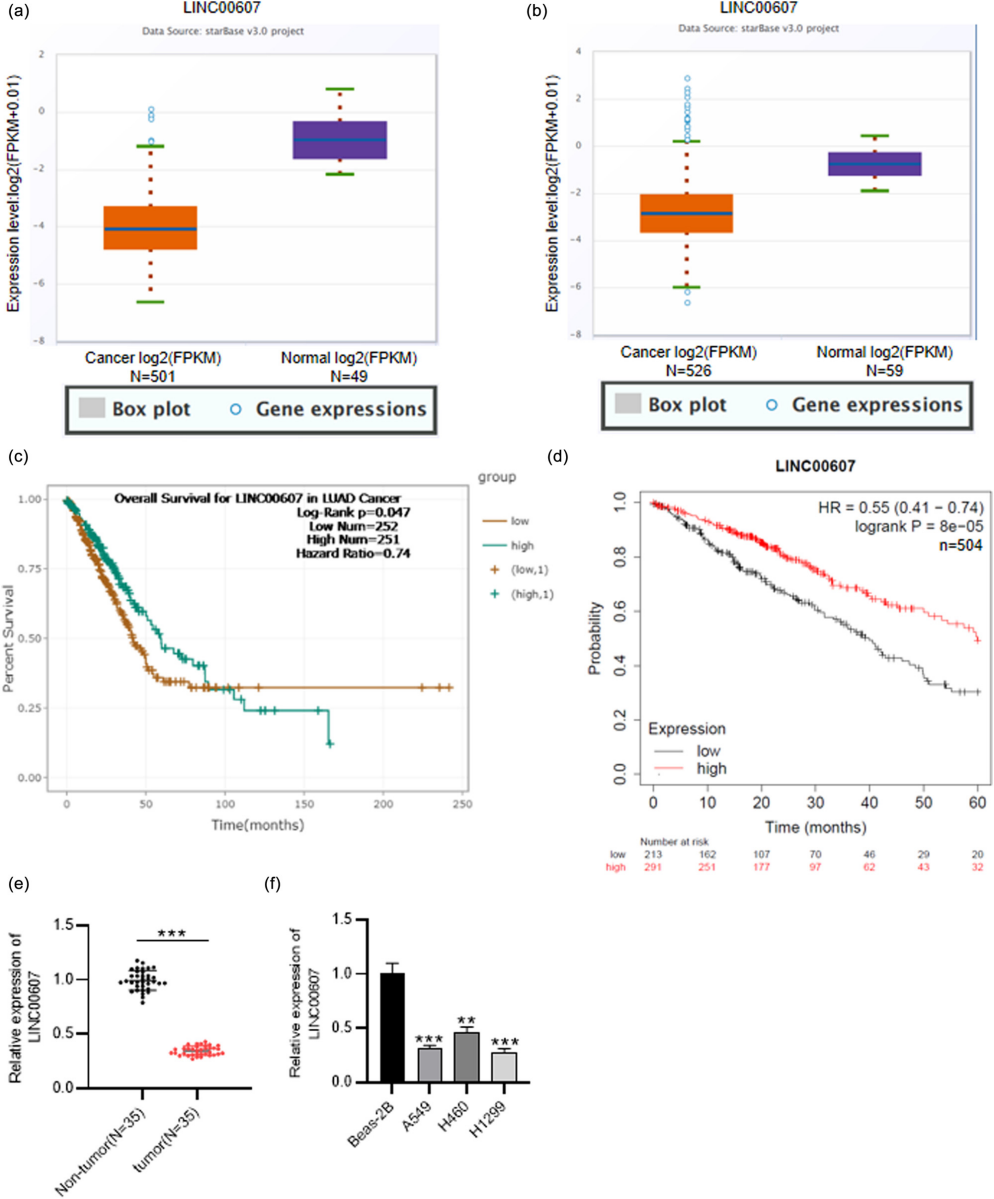
LINC00607 expression in NSCLC tissues and cells and its correlation with poor prognosis. (a) LINC00607 expression in 501 LUSC tissues compared with 49 normal tissues was predicted using ENCORI database. (b) LINC00607 level in 526 LUAD tissues versus 59 normal controls was predicted using ENCORI database. (c and d) The association between LINC00607 level and poor prognosis of LUAD or LUSC patients was predicted through ENCORI or Targetscan database. (e) RT-qPCR analysis of LINC00607 expression in 35 paired clinical NSCLC and adjacent non-tumor tissues. (f) RT-qPCR analysis of LINC00607 expression in NSCLC cells and normal cells. ^*^ *P* < 0.05, ^**^ *P* < 0.01, ^***^ *P* < 0.001.

NSCLC includes LUAD and lung squamous cell carcinoma (LUSC). Using ENCORI database (http://starbase.sysu.edu.cn/), LINC00607 was discovered to be downregulated in LUSC tissues compared with normal tissues (Figure 1a). Similarly, LINC00607 presented lower level in LUAD samples than in normal samples (Figure 1b). Low level of LINC00607 was related to poor prognosis of LAUD patients based on ENCORI database (Figure 1c). Consistently, LUSC patients with downregulated LINC00607 also had a low survival rate, as shown in the Kaplan–Meier Plotter database (https://kmplot.com/) (Figure 1d). Importantly, LINC00607 displayed lower level in clinical NSCLC tissues and NSCLC cells compared with normal controls (Figure 1e–f). In this case, we concluded that downregulated LINC00607 is correlated with the bleak prognosis of NSCLC patients.

### 3.2 Overexpressed LINC00607 inhibits NSCLC cell growth, migration, and invasion

**Figure 2:**
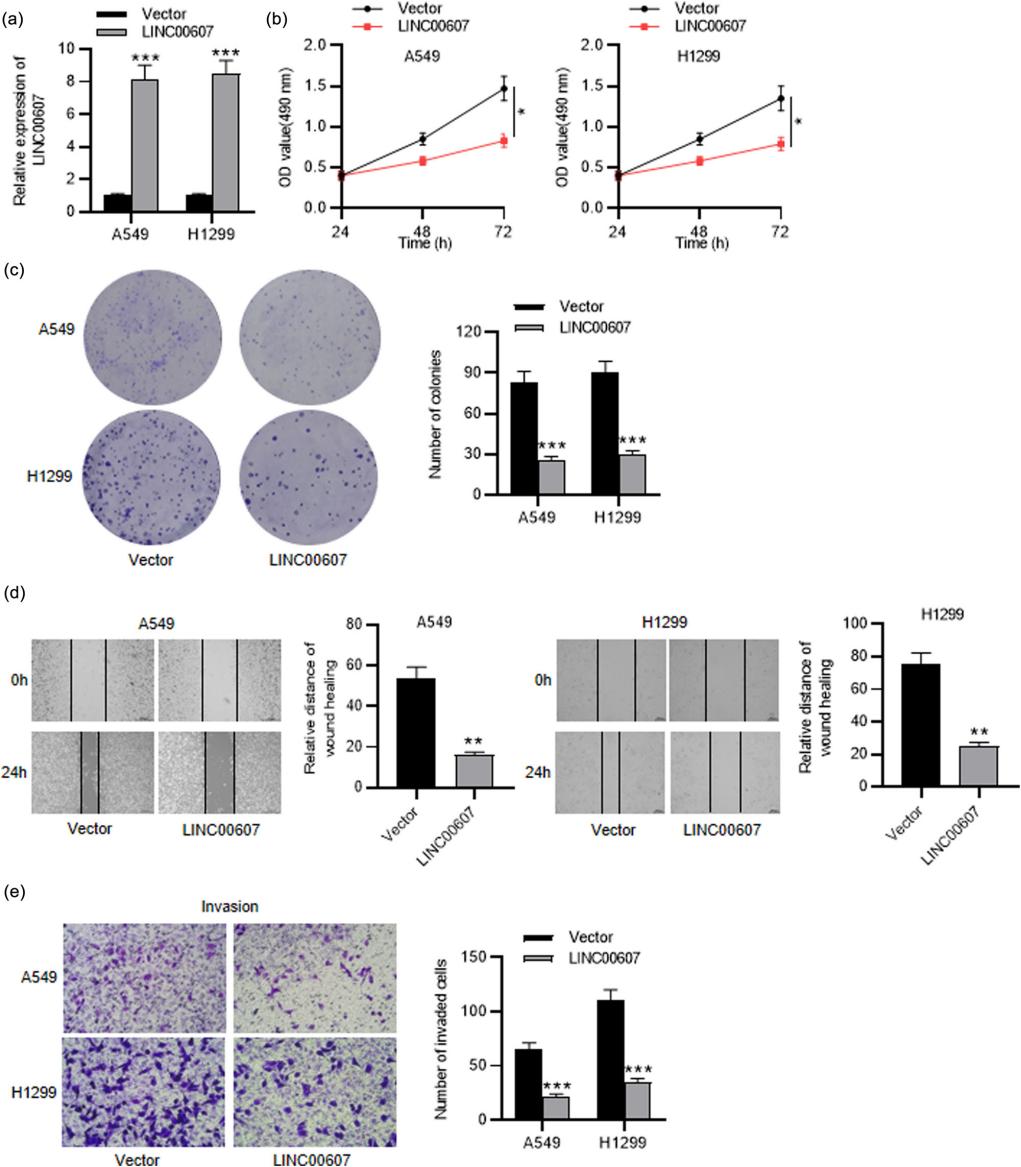
Influence of LINC00607 overexpression on NSCLC cell phenotypes. (a) RT-qPCR analysis of LINC00607 expression in NSCLC cells after LINC00607 overexpression. (b) MTT assay of NSCLC cell viability under LINC00607 overexpression. (c) Colony formation assay of NSCLC cell proliferation affected by overexpressing LINC00607. (d) Wound healing assay of NSCLC cell migration after overexpressing LINC00607. (e) Transwell assay of NSCLC cell invasion influenced by upregulated LINC00607. ^*^ *P* < 0.05, ^**^ *P* < 0.01, ^***^ *P* < 0.001.

Next, a series of functional experiments was conducted to figure out the detailed function of LINC00607 in NSCLC. RT-qPCR showed that LINC00607 was upregulated in LINC00607-overexpressed NSCLC cells (Figure 2a). First, we carried out an MTT assay to detect NSCLC cell viability under LINC00607 overexpression and found that it was repressed significantly (Figure 2b). NSCLC cell proliferation was suppressed by upregulated LINC00607, as shown in the colony formation assay (Figure 2c). Then, the wound healing assay suggested that NSCLC cell migration slowed down after overexpressing LINC00607 (Figure 2d). Additionally, NSCLC cell invasion was also inhibited by LINC00607 overexpression (Figure 2e). Thus, LINC00607 overexpression restrains NSCLC cell growth and aggressiveness.

### 3.3 LINC00607 binds with miR-1289 in NSCLC cells

**Figure 3:**
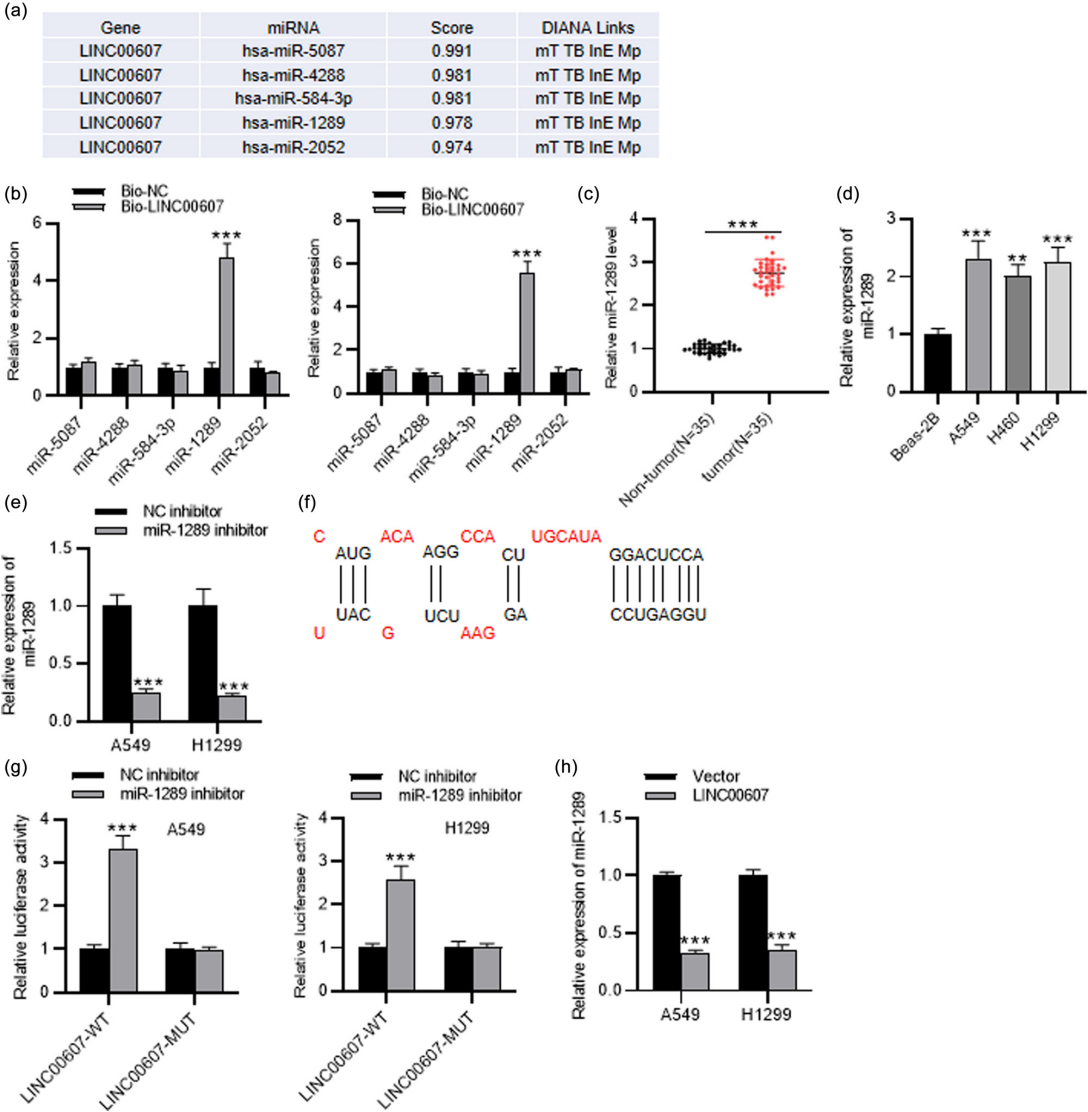
LINC00607 binds with miR-1289 in NSCLC cells. (a) Top five potential miRNAs binding to LINC00607 were predicted through DIANA database. (b) The enrichment of five potential miRNAs in Bio-LINC00607 or Bio-NC was evaluated by the RNA pull-down assay. (c) RT-qPCR analysis of miR-1289 level in NSCLC tissues (*n* = 35) and normal controls (*n* = 35). (d) RT-qPCR analysis of miR-1289 level in NSCLC cells and normal cells. (e) RT-qPCR analysis of miR-1289 level in NSCLC cells following miR-1289 downregulation. (f) The prediction of the binding site between miR-1289 and LINC00607 through DIANA database. (g) Luciferase reporter assay of the verification of the interaction between LINC00607 and miR-1289 in NSCLC cells. (h) RT-qPCR analysis of miR-1289 level in LINC00607-overexpressed NSCLC cells. ^**^ *P* < 0.01, ^***^ *P* < 0.001.

Considering that competitive binding to miRNAs is a usual mechanism for lncRNAs to perform biological functions [14], we hypothesized that LINC00607 might participate in regulating NSCLC development by competitively binding to specific miRNA. To investigate the potential miRNA targets of LINC00607, we searched in DIANA database (http://carolina.imis.athena-innovation.gr) and identified the top five candidate miRNAs containing binding sites for LINC00607 (Figure 3a). Then, RNA pull-down assay indicated that miR-1289 was highly enriched in Bio-LINC00607, while the other four did not present high enrichment in Bio-LINC00607 (Figure 3b). miR-1289 displayed higher expression in NSCLC tissues (*n* = 35) than in adjacent normal tissues (*n* = 35) (Figure 3c). Consistently, higher level of miR-1289 was also found in NSCLC cells rather than in normal cells (Figure 3d). Then, RT-qPCR revealed that the miR-1289 level was markedly decreased after miR-1289 knockdown in NSCLC cells (Figure 3e). According to DIANA database, miR-1289 binding region on LINC00607 was shown (Figure 3f). To verify their binding, we carried out the luciferase reporter assay, which suggested that the luciferase activity of LINC00607-WT was increased by miR-1289 knockdown and the effects of miR-1289 knockdown on the luciferase activity of LINC00607-MUT were negligible in NSCLC cells (Figure 3g). In addition, RT-qPCR demonstrated that the expression of miR-1289 in NSCLC cells was reduced after overexpressing LINC00607 (Figure 3h). Overall, LINC00607 binds with miR-1289 and negatively regulates its expression.

### 3.4 EFNA5 is a downstream target of miR-1289

**Figure 4:**
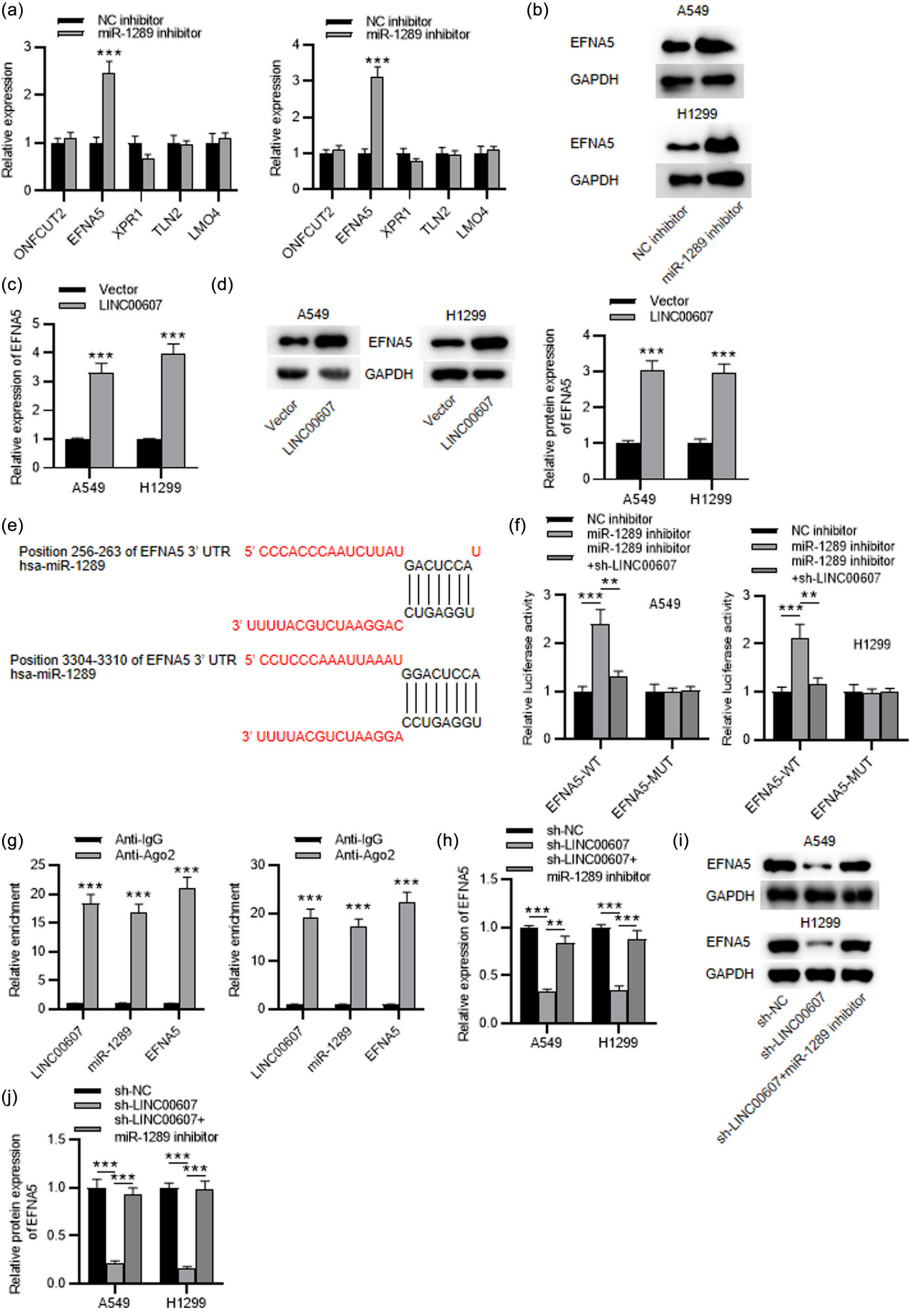
EFNA5 is a downstream target of miR-1289. (a) RT-qPCR analysis of the levels of candidate mRNAs of miR-1289 in NSCLC cells after miR-1289 knockdown. (b) Western blot analysis of EFNA5 protein level in NSCLC cells after knocking down miR-1289. (c) RT-qPCR analysis of EFNA5 mRNA expression in NSCLC cells after LINC00607 overexpression. (d) Western blot analysis of EFNA5 protein level in NSCLC cells after overexpressing LINC00607. (e) Targetscan database predicted the binding site between EFNA5 and miR-1289. (f) The luciferase activity of EFNA5-WT and EFNA5-MUT affected by miR-1289 inhibition and LINC00607 silencing in NSCLC cells was assessed by the luciferase reporter assay. (g) The enrichment of LINC00607, miR-1289, and EFNA5 in anti-Ago2 and control anti-IgG groups was examined by RIP assay. (h–j) RT-qPCR and western blot analyses of EFNA5 in NSCLC cells after LINC00607 silencing and miR-1289 knockdown. ^**^ *P* < 0.01, ^***^ *P* < 0.001.

Using miRDB database (http://mirdb.org/), we selected top five potential mRNAs binding to miR-1289 (Table S1). Their expression was measured in miR-1289 inhibitor- or NC inhibitor-transfected NSCLC cells by RT-qPCR, which revealed that EFNA5 mRNA expression was significantly increased by miR-1289 knockdown (Figure 4a). EFNA5 protein level was also higher in miR-1289-downregulated NSCLC cells (Figure 4b). Furthermore, overexpression of LINC00607 remarkably inhibited the mRNA and protein expression of EFNA5 in NSCLC cells (Figure 4c–d). The potential binding region of miR-1289 on EFNA5 was discovered through Targetscan database (http://www.targetscan.org/) (Figure 4e). Subsequently, the relationship between LINC00607, miR-1289, and EFNA5 was explored by the luciferase reporter assay and RIP assay. According to the luciferase reporter assay, the luciferase activity of EFNA5-WT was markedly enhanced by miR-1289 downregulation and the enhancement was offset by LINC00607 silencing in NSCLC cells (Figure 4f). In addition, RIP assay illustrated that LINC00607, miR-1289, and EFNA5 were all significantly enriched in the anti-Ago2 group, indicating the coexistence of LINC00607, miR-1289, and EFNA5 in RNA-induced silence complexes (Figure 4g). Finally, we discovered that the inhibition of miR-1289 reversed the reduction in EFNA5 mRNA and protein expression caused by LINC00607 silencing in NSCSL cells (Figure 4h–j). Therefore, LINC00607 binds with miR-1289 to attenuate the suppression of miR-1289 on the expression of EFNA5.

### 3.5 EFNA5 is downregulated in NSCLC tissues and cells and is correlated with lower survival rate of NSCLC patients

**Figure 5:**
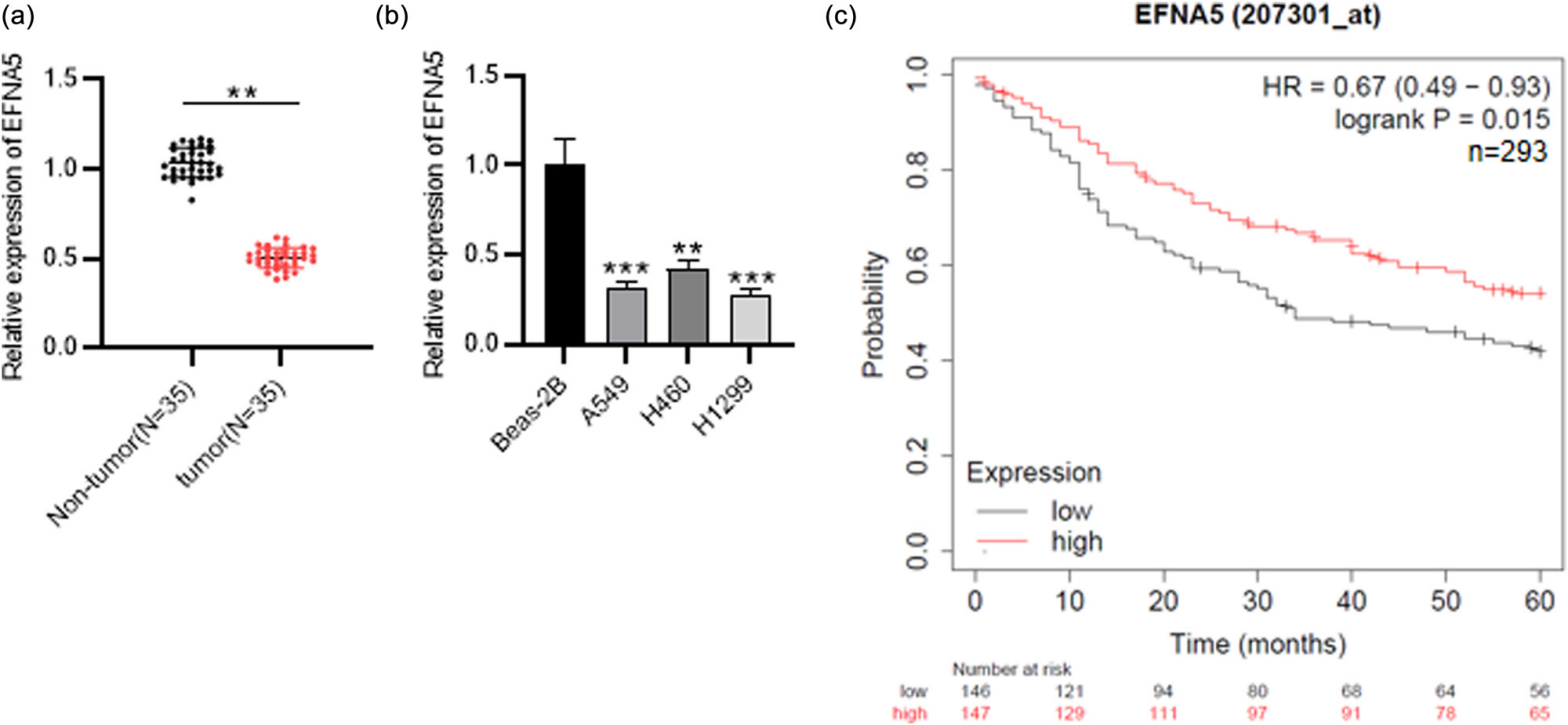
EFNA5 expression NSCLC and its association with poor prognosis. (a) RT-qPCR analysis of EFNA5 level in 35 paired NSCLC tissues and normal tissues. (b) RT-qPCR analysis of EFNA5 level in NSCLC cells and normal cells. (c) The correlation between the prognosis of NSCLC patients and EFNA5 level was analyzed through the Kaplan–Meier Plotter database. ^**^ *P* < 0.01, ^***^ *P* < 0.001.

Subsequently, whether EFNA5 is aberrantly expressed in NSCLC cells was assessed. First, EFNA5 level in NSCLC tissues (*n* = 35) was lower than in adjacent normal tissues (*n* = 35), suggested by the results of RT-qPCR (Figure 5a). EFNA5 downregulation was also observed in NSCLC cells versus normal cells (Figure 5b). Furthermore, as shown in the Kaplan–Meier Plotter database, the bleak prognosis of NSCLC patients was associated with low expression of EFNA5 (Figure 5c). Thus, downregulated EFNA5 in NSCLC is associated with poor prognosis of NSCLC patients.

### 3.6 Overexpressed EFNA5 suppresses NSCLC cell growth, migration, and invasion

**Figure 6:**
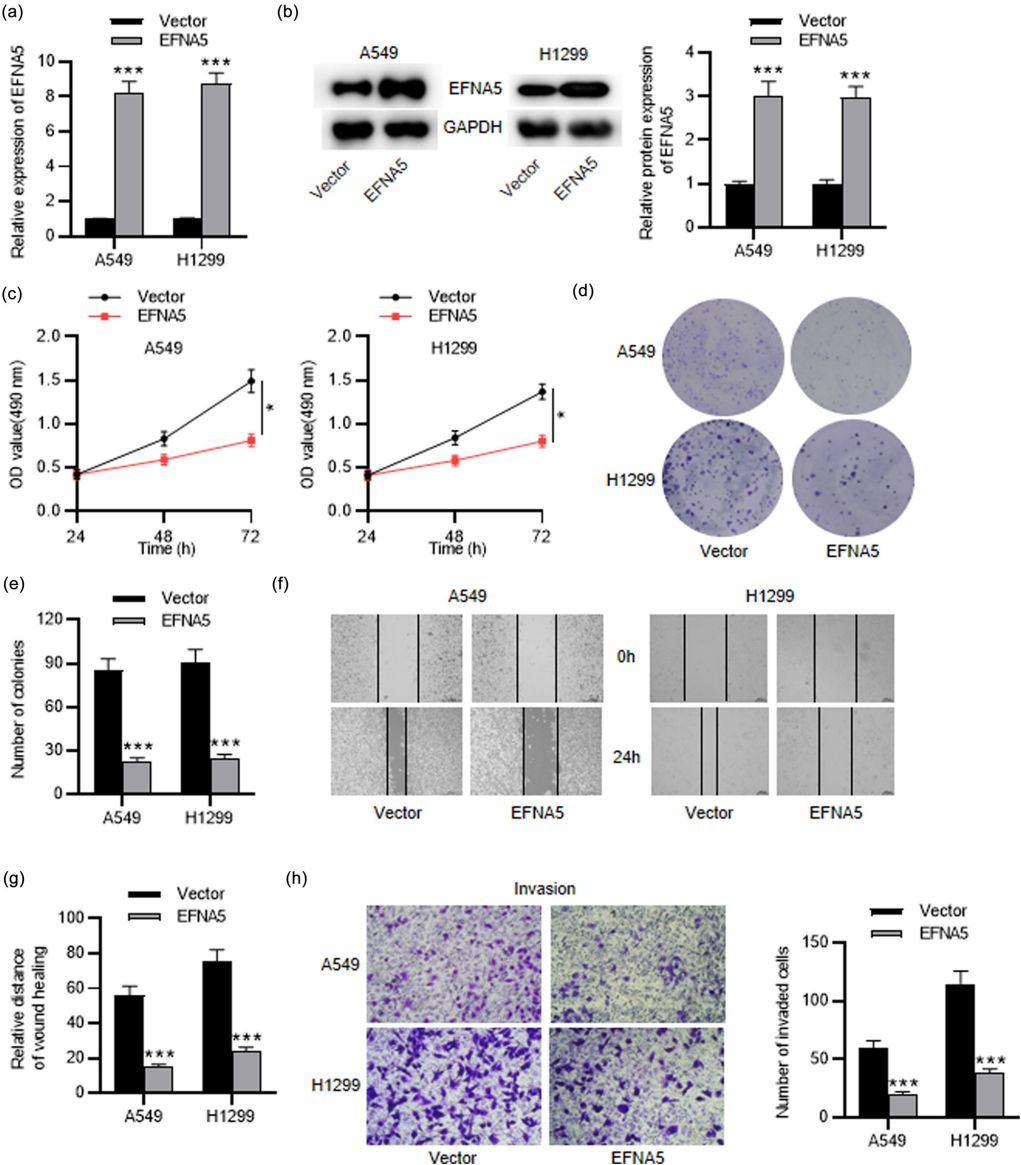
The influence of EFNA5 overexpression on NSCLC cell phenotypes. (a) RT-qPCR analysis of EFNA5 mRNA expression in NSCLC cells after EFNA5 overexpression. (b) Western blot analysis of EFNA5 protein level in NSCLC cells after overexpressing EFNA5. (c) MTT assay of NSCLC cell viability under EFNA5 overexpression. (d and e) Colony formation assay of NSCLC cell proliferation affected by overexpressing EFNA5. (f and g) Wound healing assay of NSCLC cell migration after overexpressing EFNA5. (h) Transwell assay of NSCLC cell invasion influenced by upregulated EFNA5. ^*^ *P* < 0.05, ^***^ *P* < 0.001.

To investigate the influence of EFNA5 on the malignant phenotypes of NSCLC cells, we conducted a series of gain-of-function experiments. First, the overexpression efficiency of EFNA5 was evaluated by RT-qPCR and western blot analyses, which indicated that both mRNA and protein levels of EFNA5 were elevated in EFNA5-overexpressed NSCLC cells (Figure 6a and b). Then, as shown by the MTT assay, the viability of NSCLC cells was considerably attenuated after overexpressing EFNA5 (Figure 6c). Colony formation assay demonstrated that overexpression of EFNA5 greatly repressed NSCLC cell proliferation (Figure 6d and e). Moreover, the migratory and invasive capabilities of EFNA5-overexpressed NSCLC cells were discovered through the wound healing assay and Transwell invasion assay (Figure 6f–h). In summary, overexpressed EFNA5 inhibited the malignant behaviors of NSCLC cells.

### 3.7 EFNA5 knockdown reverses the effects of LINC00607 overexpression on NSCLC cell phenotypes

**Figure 7:**
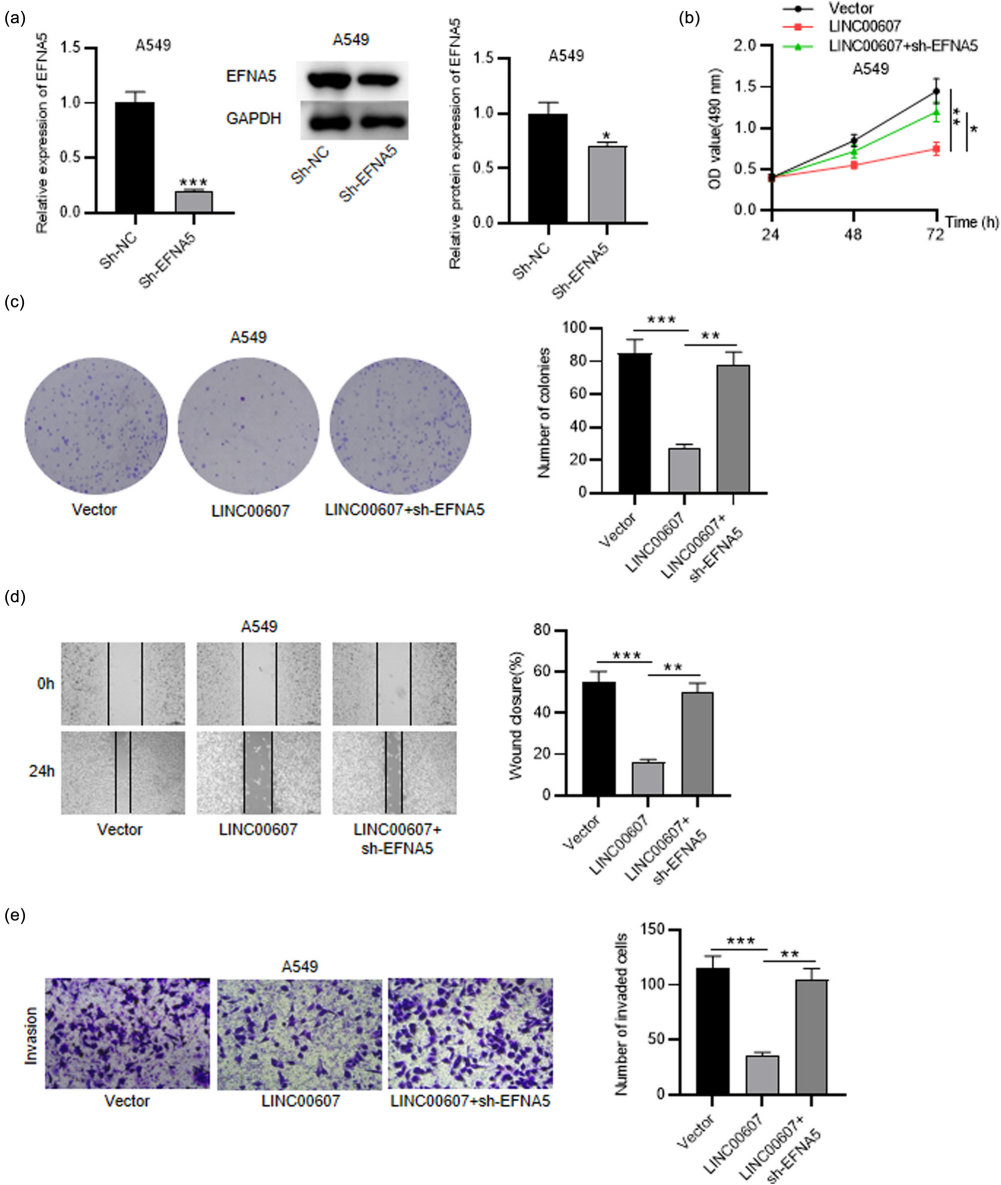
EFNA5 knockdown antagonizes the influence of LINC00607 on NSCLC cell phenotypes. (a) RT-qPCR and western blot analyses of EFNA5 level in NSCLC cells after downregulating EFNA5. (b) MTT assay of NSCLC cell viability in each group. (c) Colony formation assay of cell proliferative ability in each group. (d) Wound healing assay of NSCLC cell migration in each group. (e) Transwell assay of NSCLC cell invasive capability in each group. ^*^ *P* < 0.05, ^**^ *P* < 0.01, ^***^ *P* < 0.001.

To elucidate how LINC00607-involved regulatory mechanism affects NSCLC cell phenotypes, we performed several rescue experiments. First, EFNA5 was knocked down in NSCLC cells after transfection with sh-EFNA5 (Figure 7a). MTT showed that the repression of LINC00607 overexpression in cell viability was neutralized by EFNA5 knockdown in NSCLC cells (Figure 7b). Similarly, the repressed NSCLC cell proliferative ability under LINC00607 upregulation was restored by EFNA5 silencing, as shown in the colony formation assay (Figure 7c). Additionally, the depletion of EFNA5 countervailed the suppressive effects of overexpressing LINC00607 on NSCLC cell migration (Figure 7d). The silencing of EFNA5 reversed the NSCLC cell invasive ability weakened by LINC00607 overexpression, as presented in the Transwell assay (Figure 7e). Therefore, we concluded that LINC00607 overexpression-inhibited NSCLC cell proliferation and aggressiveness was countervailed by EFNA5 knockdown.

## 4 Discussion

NSCLC remains a challenging malignancy with a 5-year survival rate of less than 20%, despite innovative treatments that have been introduced over the past decade [15]. Accumulating studies have suggested that lncRNAs play pivotal roles in mediating multiple biological processes such as tumor cell growth and metastasis [16]. In this study, we innovatively illustrated that LINC00607 was markedly downregulated in NSCLC, and LINC00607 suppressed NSCLC cell growth, migration, and invasion via modulating the miR-1289/EFNA5 axis, which indicated that LINC00607 functioned as a tumor suppressor in NSCLC.

Increasing evidence suggests that lncRNAs exert oncogenic or tumor-suppressive role via serving as miRNA sponges to modulate mRNA expression [17,18]. Bioinformatics analysis showed that miR-1289 contained a binding site for LINC00607, and the interaction was verified through RNA pull-down assay and luciferase reporter assay. In fact, the abnormal expression of miR-1289 was observed in different tissues. The aberrant level of miR-1289 was detected in hepatocellular carcinoma specimens [19]. miR-1289 expression was significantly elevated in gastric biopsies from patients infected with cagA (+) *Helicobacter pylori* [20]. Herein, we discovered that miR-1289 was overexpressed in NSCLC cells and tissues. Thus, LINC00607 played a suppressive role in NSCLC through binding with miR-1289 and regulating the target genes.

We continued to explore the potential target genes that can bind to miR-1289 and EFNA5 was identified as its expression and protein levels were significantly increased under miR-1289 knockdown. Previously, EFNA5 was frequently reported as a target gene in various cancers. For example, EFNA5 is targeted by miR-645 to promote cell proliferation in colorectal cancer [21]. EFNA5, as a target of PIWI (P element-induced wimpy testis) integrating RNA (piRNA) piR-017061, is regulated by PIWIL1 to facilitate pancreatic cancer progression [22]. However, EFNA5 was never found to be targeted by miRNAs and play a role in NSCLC. Herein, we first explored and confirmed the binding between EFNA5 and miR-1289 in NSCLC. The expression of EFNA5 was discovered to be negatively regulated by miR-1289 and positively regulated by LINC00607. Importantly, luciferase reporter assay and RIP assay validated the interactions between LINC00607, miR-1289, and EFNA5. Thus, we concluded that LINC00607 regulated EFNA5 expression by sponging miR-1289. Furthermore, EFNA5 presented lower expression in NSCLC cells and tissues, and downregulated EFNA5 was confirmed to be correlated with the lower survival rate of NSCLC patients. Subsequently, a series of gain-of-function experiments were performed to evaluate the effects of EFNA5 on NSCLC cell phenotypes. The results showed that EFNA5 overexpression markedly inhibited NSCLC cell viability, proliferation, migration, and invasion. Additionally, we knocked down EFNA5 in LINC00607-overexpressed NSCLC cells to explore its role in LINC00607-associated regulatory mechanism in NSCLC cells. We found that silencing of EFNA5 countervailed LINC00607 overexpression-repressed cell growth and aggressiveness in NSCLC. Therefore, we inferred that LINC00607 inhibited NSCLC progression by completely binding to miR-1289 and modulating EFNA5 level.

In summary, we demonstrated that LINC00607 was downregulated in NSCLC and LINC00607 downregulation was related to poor prognosis of NSCLC patients. LINC00607 was identified as an antioncogene in NSCLC by sponging miR-1289 and regulating EFNA5 expression in NSCLC. We are the first to attach importance to the regulatory role of the LINC00607/miR-1289/EFNA5 axis in NSCLC, which may yield an important insight into the methods of NSCLC diagnosis and therapy.

## Supplementary Material

Supplementary material
